# Probing the Ion Binding Site in a DNA Holliday Junction Using Förster Resonance Energy Transfer (FRET)

**DOI:** 10.3390/ijms17030366

**Published:** 2016-03-10

**Authors:** Jacob L. Litke, Yan Li, Laura M. Nocka, Ishita Mukerji

**Affiliations:** Department of Molecular Biology and Biochemistry and Molecular Biophysics Program, Wesleyan University, Middletown, CT 06459-0175, USA; jlitke@wesleyan.edu (J.L.L.); lyan01@wesleyan.edu (Y.L.); lnocka@berkeley.edu (L.M.N.)

**Keywords:** Holliday junctions, nucleic acids, FRET, ion-binding, lanthanide luminescence

## Abstract

Holliday Junctions are critical DNA intermediates central to double strand break repair and homologous recombination. The junctions can adopt two general forms: open and stacked-X, which are induced by protein or ion binding. In this work, fluorescence spectroscopy, metal ion luminescence and thermodynamic measurements are used to elucidate the ion binding site and the mechanism of junction conformational change. Förster resonance energy transfer measurements of end-labeled junctions monitored junction conformation and ion binding affinity, and reported higher affinities for multi-valent ions. Thermodynamic measurements provided evidence for two classes of binding sites. The higher affinity ion-binding interaction is an enthalpy driven process with an apparent stoichiometry of 2.1 ± 0.2. As revealed by Eu^3+^ luminescence, this binding class is homogeneous, and results in slight dehydration of the ion with one direct coordination site to the junction. Luminescence resonance energy transfer experiments confirmed the presence of two ions and indicated they are 6–7 Å apart. These findings are in good agreement with previous molecular dynamics simulations, which identified two symmetrical regions of high ion density in the center of stacked junctions. These results support a model in which site-specific binding of two ions in close proximity is required for folding of DNA Holliday junctions into the stacked-X conformation.

## 1. Introduction

Holliday Junctions are composed of four DNA strands and are central to the process of homologous recombination. This process contributes to genetic and biological diversity, by promoting new varieties of gene combinations in chromosomes through gene transfer mechanisms in bacteria and crossover events in meiosis [1]. Short inverted repeats, which are needed to form four way junction (4WJ) *in vivo*, induce genetic instability and may play a role in promoting mutations that lead to cancer and other diseases [2]. Holliday Junctions are flexible, dynamic structures that can exist in two conformations, open and stacked, where the open state facilitates branch migration and the exchange of genetic material. The stacked conformation is induced through the binding of metal cations, regardless of valency, and by interactions with proteins such as T4 Nuclease VII, Hjc and RusA while proteins such as RuvABC and λ Integrase stabilize the open conformation [3]. Junctions are central intermediates in double-strand DNA break repair, a process important for maintaining chromosome stability. DNA junctions have also been utilized in nanotechnology, as modulation of the open and stacked forms provides the basis for a biomolecular switch [4]. Thus, elucidating the mechanism of this open-stacked conformational transition is important for understanding the behavior of junctions in native or synthetic systems.

Previous *in vitro* studies of four-way junction structures have focused on synthetically assembled junctions, consisting of non-homologous sequences creating an immobile junction. Early studies using restriction digest enzymes identified stacked conformations in high salt concentrations and open conformations in low salt by the relative gel mobility patterns of the digestion products [5]. Along with evidence from birefringence, neutron scattering, and hydroxyl radical probing, this work confirmed an anti-parallel orientation of non-crossing junction strands in the stacked structure [6,7]. There are two possible conformers associated with the anti-parallel orientation of arms in the stacked structure, isoI and isoII, and changes in sequence in the junction core alter the relative conformer populations in the stacked conformation [5,8]. Four way junction structures have also been extensively characterized using X-ray crystallography [3,9,10]. In addition, protein-junction X-ray structures have furthered understanding of the different conformations. In these determinations, the multiple crystal structures of four way junctions have identified some structural-effects of sequence and suggested an ACC trinucleotide pattern stabilizes Holliday junctions in a stacked conformation [11,12,13].

Förster resonance energy transfer (FRET) studies have been used successfully to study junction structure and protein-junction interactions. In these studies, fluorophore pairs were placed on neighboring arms at the 5′ ends and the relative proximity was determined by FRET [14,15,16]. These initial studies determined the properties of anti-parallel non-crossing strands, sequence preference among stacked conformations and the relative population of the different conformers, which was subsequently confirmed with single molecule measurements [14,17,18]. In the current study, we used the relatively well-characterized junction 3 originally developed by Lilley and coworkers, to examine ion binding.

Divalent cations mediate the open to stacked-X transition and Mg^2+^ concentrations *in vivo* are likely to lead to binding and formation of a stacked junction. Several other ions have also been observed to induce this conformational change. Osmium tetraoxide reactivity assays with thymine bases have pointed to the binding of ions in the central region of the junction [19]. Uranyl photoprobing experiments have also established that ion binding occurs in the center of the junction and is needed for junction stacking [20]. Divalent ions are more effective at inducing the stacked structure and completely fold the junction at much lower concentrations (~100 μM) relative to monovalent ions (~40 mM), while trivalent cobalt hexaamine was observed to fold junctions at concentrations as low as 2 μM [19]. The increased efficiency of multivalent ions to modulate junction conformation is attributed to their increased charge density and greater ability to neutralize charge within the junction core. This hypothesis has been borne out by recent crystal structures of stacked junctions in the presence of different ion combinations, which indicate the presence of ions in the junction core [10,21]. Despite the significant effect of metal cations on junction structure, the nature of the ion interaction needed for folding the junction into the stacked-X conformation is not completely known. We expect that these ions are mobile and exchanging with the bulk solution, but do experience longer residence times in the center of the junction to facilitate adoption of the stacked-X form. A combined molecular dynamics and single molecule study has suggested that the adoption of distinct states arises from metal ions binding to loops in the center of the junction [22]. Molecular dynamics simulations of a different junction have indicated two regions of high ion density exist in the central region of the junction in symmetrical positions [23]. Experimentally, the number of ions needed to modulate junction structure is not known. Although adoption of the stacked form was shown to require ion binding, details of the ion environment, particularly if direct coordination to the phosphate backbone is needed, have yet to be determined. In this study we have used FRET, lanthanide luminescence and ITC measurements to explore ion coordination, ion-induced conformational changes and the ion-binding site. These measurements have demonstrated that the ion-mediated folding reaction requires site-specific binding of two ions, which are located within 7 Å of each other and the interaction involved at least one site of direct coordination to the backbone.

## 2. Results and Discussion

We have explored the ability of different metal ions to induce a conformational change of a DNA four-way junction (4WJ). The junction used for this study, junction 3, was originally developed by Lilley and coworkers [5], is not capable of branch migration, and has been well-characterized by equilibrium FRET and single molecule experiments [14,17,18]. The wealth of information known about this junction makes it a good model system for our study.

### 2.1. FRET Measurements of Ion Affinities and Junction Folding

**Figure 1:**
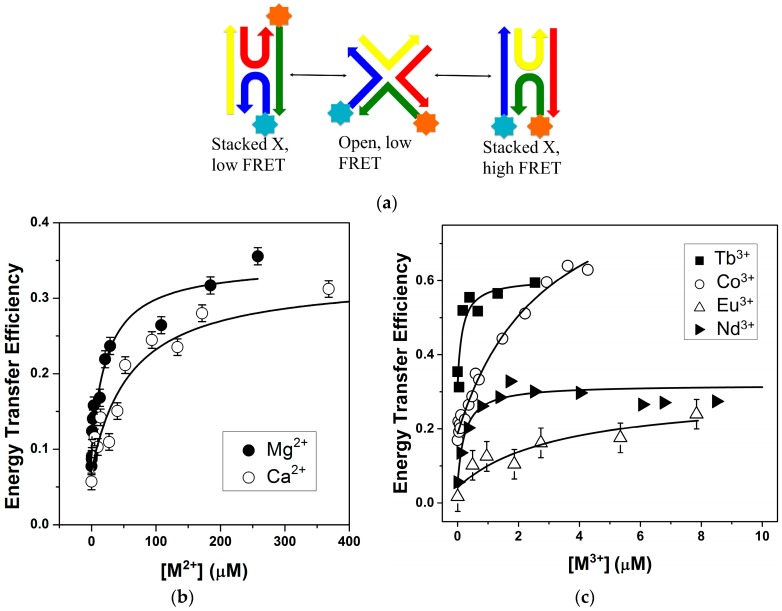
Increasing multivalent cation concentration folds the junction bringing the labeled arms of the DNA closer together and increases transfer efficiency. (**a**) Schematic of the junction folding that is induced by ion binding. The R and X strands have a linker at the 5′ end for attachment of the fluorescein (FAM) and tetramethylrhodamine (TAMRA) dyes, which are in close proximity in the high FRET, isoII conformation; (**b**) Fitting of binding curves yielded apparent *K*_d_ values of 55 ± 19 and 22 ± 8 μM for Ca^2+^ and Mg^2+^, respectively; (**c**) Fitting of binding curves yielded apparent *K*_d_ values of 4 ± 0.3, 0.1 ± 0.1, 3 ± 2 and 0.7 ± 0.3 µM for [Co(NH_3_)_6_]^3+^, Tb^3+^, Eu^3+^ and Nd^3+^, respectively. Titrations of cobalt hexamine contained 5 mM Na^+^, while all other titrations contained only 0.5 mM Na^+^. For clarity of presentation, the Co^3+^ and Tb^3+^ data are offset from the y-axis by +0.15 and +0.3, respectively, and only the error associated with the Eu^3+^ data is shown. The other ion binding curves have similar error and error bars. All titrations were performed at 10 °C in a 10 mM Tris–HCl pH 7.4 buffer.

The ability of ions to fold the junction into a stacked conformation was monitored by FRET in which the 5′ ends of junction arms R and X (see Figure 1) were labeled through a six-carbon linker with donor and acceptor dyes. Upon addition of metal ions, two junction arms stack on top of each other to form a quasi-continuous helix. In the case of junction 3 this stacking brings the R and X arms closer together increasing the FRET efficiency [14,16].

As expected, for all of the ions investigated (Figure 1a,b), an increase in ion concentration led to an increase in energy transfer, which plateaued upon saturation. The initial energy transfer efficiency was around 0.05, consistent with high concentrations of an open junction where the arms are too far apart for efficient transfer. As shown in Figure 1, the junction can exist in two stacked populations, isoI or in our case the low FRET state and isoII, the high FRET state. For the efficiency analysis, we have assumed that the population of the high FRET conformer is 77% based upon populations determined in the gel and by fluorescence using single molecule and equilibrium energy transfer methods [14,17,18]. These previous measurements were done with a number of different ion types at different concentrations and did not observe any changes in relative population. Conformer distributions have not been previously reported for Ln(III) ions, but different ion types are not expected to induce a change in these relative populations. Previous NMR and fluorescence measurements characterizing junction sequence and conformation have illustrated that the bases at the center predominantly determined junction conformation [8].

The addition of ions largely led to comparable changes in junction conformation, with a change in energy transfer efficiency of 0.25–0.35. These transfer efficiencies yielded distances between 50–60 Å and an associated junction interduplex angle of 54°–60° (see Supplementary Material, Table S1) [24,25]. Energy transfer is not observed for the isoI conformation as the distance between the dyes is estimated to be over 110 Å, which is longer than 2*R*_0_ for the dye pair used. The effect of the relative dye position on the helix axis on the measured distance is approximately 8 Å (Supplementary Material, Figure S1), which is smaller than the error introduced from the relative mobility of the dyes (±11 Å). The relative mobility is estimated from the steady state anisotropy values of the dyes in the donor only and acceptor only junctions. The relative proximity of the R and X junction arms, as indicated by the interduplex angle (IDA), was roughly proportional to the ionic radius within a particular ion type (monovalent or polyvalent) (Supplementary Material, Figure S1) [26]. Although the observed changes were small relative to the error range of the distance determination, relative changes in FRET efficiency can be compared. The correlation between ion type and efficiency or IDA is consistent with prior work that suggested junction stacking is achieved through ion binding at the center [19,20,21]. The current FRET measurements have further suggested that ion size potentially also influences the amount of ion-induced conformational change. This dependence of transfer efficiency on ion type is consistent with a model where ion binding is occurring at a specific site(s) within the junction and extends earlier findings [14].

A two-state binding model of open and stacked junctions was used to determine the ion concentration needed for junction stacking, (See Section 3.3). Although other binding models can be used to describe the data (Supplementary Material, Figure S2), we employed this two-state model as the ion-binding site for the junction stacking interaction is not well defined and the number of ions needed is not known. Moreover, this model has been used successfully to describe ion binding to junctions [14,16] and more generally, ions binding to nucleic acids [27]. The ion concentration where the stacked and open populations of the junction were equal was used to define an apparent dissociation constant (*K*_d_). We observed that the charge density of the ion largely determines the apparent affinity. In the case of monovalent ions, the apparent *K*_d_ was in the millimolar range, whereas the polyvalent ions yielded *K*_d_ values in the µM range. Interestingly, we also found that the apparent *K*_d_ values for the trivalent ions were approximately 10–20-fold lower than those measured for the divalent ions. (Supplementary Material, Table S1) The greater effectiveness of the trivalent ions in inducing formation of the stacked conformation is consistent with their higher charge density. In all cases, the ion binding was non-cooperative, with Hill coefficients in the 1–1.1 range. In this instance, the Hill coefficient describes a two-state equilibrium in which the folded state has n more ions than the unfolded state [27]. As the junctions were in a solution containing Na^+^, the ion-induced formation of the stacked junction necessarily involved the displacement of ions to maintain charge neutrality. Thus, the Hill coefficient reflects the net change in ion binding as a consequence of the conformational change rather than the number of ions needed to effect the change. In the stacked conformation, the negatively charged backbones are in close proximity, creating an electronegative cleft, and the positively charged ions are needed to aid in the neutralization of the phosphate backbone and reduce repulsion [10,19,20,21].

### 2.2. Thermodynamic Parameters of Ion Binding and Stoichiometry

**Figure 2:**
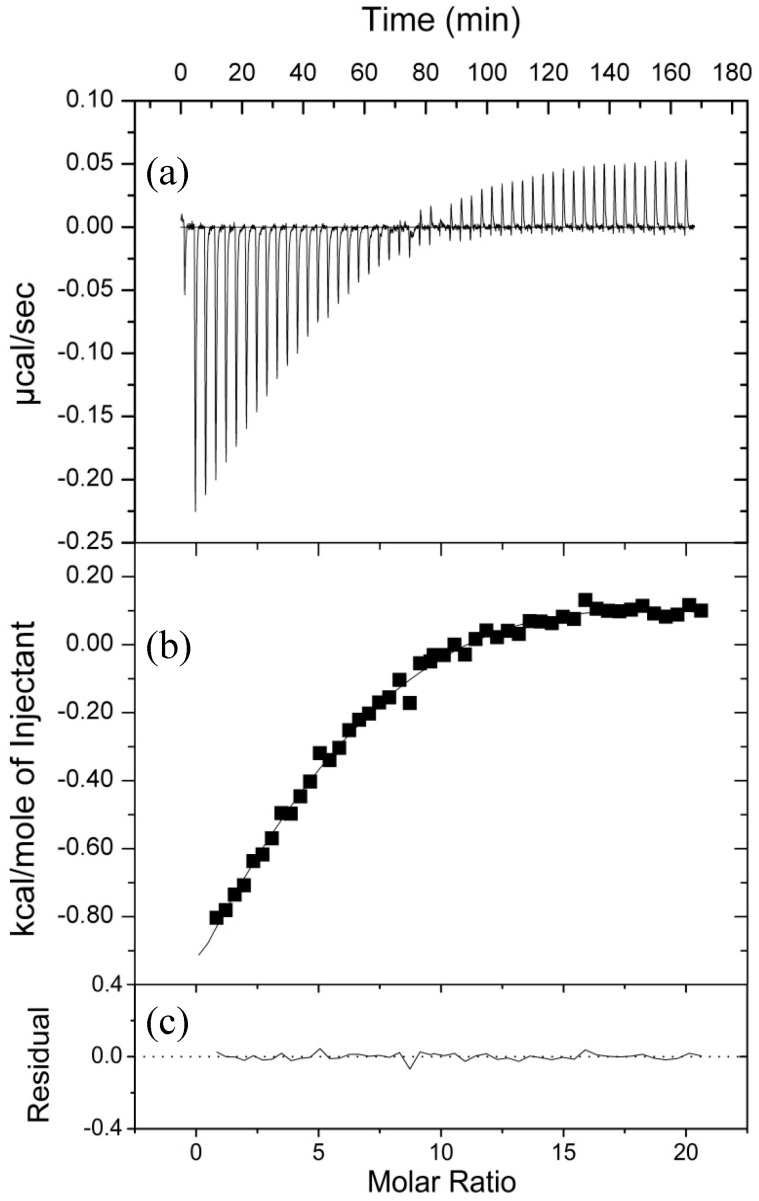
Mg^2+^ binding to the DNA 4WJ as measured by isothermal titration calorimetry. (**a**) Raw data and (**b**) normalized integration of the raw data for the titration of 0.85 mM Mg^2+^ into a 16 μM solution of DNA 4WJ; (**c**) Residuals depicted are generated between the actual data and the fit shown. Both solutions contained 5 μM EDTA, 5 mM Na^+^, and 10 mM Tris–HCl pH 7.4. Two titrations of 25 × 10-μL injections were performed and appended using the Concat32 software (MicroCal of Malvern Instruments Ltd., Worchestershire, UK).

We used isothermal titration calorimetry (ITC) to further probe the nature of ion binding. In these experiments, Mg^2+^ was titrated into a solution of the 4WJ and the accompanying changes in enthalpy associated with ion binding were analyzed in terms of thermodynamic parameters. In this case, ∆*H* changes are associated with the making and breaking of bonds while ∆*S* changes are associated with solvent reorganization and conformational changes. To sample a broad concentration range, two titrations of 25 injections each were concatenated for the final results. As shown in Figure 2, Mg^2+^ binding led to an initial exothermic reaction followed by an endothermic reaction. Initial analysis of the ITC data revealed that a single class of ion binding site was not able to accurately describe the exothermic and endothermic changes observed over the course of the titration and did not describe the data well, by the statistical parameters of the fit or by eye.

**Figure 3:**
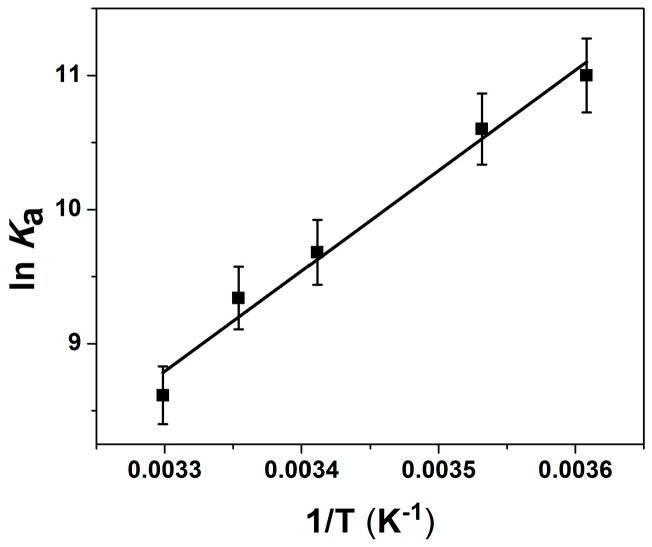
Van’t Hoff Analysis of Ion binding to the DNA 4WJ. The *K*_a_ values were determined from temperature dependent FRET experiments that monitored junction conformation upon ion binding (Supplementary Material, Figure S2). Titrations were carried out in a solution of 50 nM 4WJ, 0.5 mM Na^+^ and 10 mM Tris–HCl with pH 7.4.

The interdependency of the thermodynamic parameters led to difficulties in resolving more than one binding event from the ITC data alone. Since the FRET experiments only report on the effect of ion binding on junction conformation, we elected to measure the parameters associated with the ion-induced conformational changes using the van’t Hoff relationship. It has been shown previously, that thermodynamic parameters determined from a van’t Hoff analysis agree well with ITC-determined values [28]. FRET experiments were performed at five different temperatures (Supplementary Material, Figure S4) and *K*_a_ values were determined at each temperature as described above. The van’t Hoff determined (Figure 3) enthalpy and entropy values associated with ion binding and the FRET-measured conformational changes are given in Table 1. The independently determined parameters of *K*_a_ and ∆*H* were used in the analysis of the ITC data, which yielded a more robust fit with fewer variable parameters. Chi-squared values and residuals were markedly improved with the addition of a second class of binding site. The binding events were described in terms of: (1) a strong exothermic (−14.9 kcal/mol) interaction, with a negative entropy, and a stoichiometry of 2; and (2) a weaker endothermic interaction (1.9 kcal/mol), accompanied by a positive entropy change, and a stoichiometry of ~16.

We have attributed the exothermic reaction, as measured by FRET to ions binding to the center of the junction and inducing the stacked conformation. Increased stacking interactions of the bases and new hydrogen bonding patterns are expected to result from the ion-induced change in conformation and contribute to the negative enthalpy change observed. The decrease in entropy is attributed to the relative ordering of junction arms into the stacked conformation as well as to ions binding to the junction center. The stoichiometry of this exothermic interaction suggested that two ions are needed for specific folding of the junction, which was in good agreement with crystal structures of the junction and molecular dynamics simulations [21,23]. The exothermic nature of the binding is also attributed to water-mediated interactions between the ions and the backbone with the possibility of at least one direct contact between ion and backbone as discussed below.

The weaker, positive endothermic reaction is ascribed to the displacement of Na^+^ ions from the condensed layer of ions surrounding the backbone. The experiments were done in a concentration of 5 mM Na^+^, which was sufficient for neutralization of the DNA but not enough to stabilize the junction in the stacked conformation [16]. At sufficiently high concentrations of Mg^2+^, the Mg^2+^ can displace the condensed counterions, resulting in an entropic driving force for Mg^2+^ association, as expected [29]. The stoichiometry for this binding reaction was 15.9 ± 2.0 suggesting that at the highest concentration of Mg^2+^ used in this study, the condensed layer remains one that contains both Na^+^ and Mg^2+^ ions. Similar entropically driven, but enthalpically opposed (∆*H* ~+1 kcal/mol) reactions have been previously observed for cation binding to plasmid DNA by ITC and supports the assignment of this second class of interaction to non-specific ion binding to the backbone [30].

### 2.3. Lanthanide Luminescence and Multiplicity of Binding Sites

To directly explore the nature of the ion binding site(s), we turned to luminescence experiments with lanthanide ions. Lanthanide(III) ions have been shown to be able to replace Mg^2+^ in folding of nucleic acids and the mode of ion binding is presumed to be similar [31]. Although the ionic radii are different (0.86 *vs.* 1.20 Å for Mg^2+^ and Eu^3+^, respectively) the oxophilicity and hard base character of the lanthanides as well as the large hydration energies make them good substitutes for Mg^2+^ [31,32]. Since Ln(III) binding led to similar changes in transfer efficiency and hence, junction conformation as Mg^2+^ (Figure 1), we considered that the Ln(III) ions were binding to the same or similar sites as the Mg^2+^. One of the main advantages of using lanthanide luminescence to probe ion binding arises from the existence of non-degenerate electronic transitions. The energies of these transitions reflect the direct coordination environment of the ion and the nature of the associated ligands [33].

**Figure 4:**
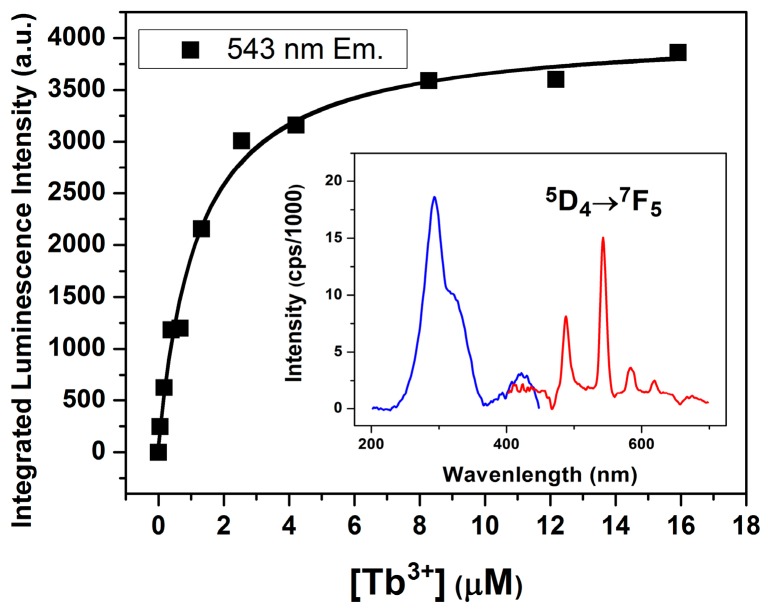
Tb^3+^ binding to the DNA 4WJ measured by luminescence. Titration of TbCl_3_ into 10 µM junction leads to an increase in Tb^3+^ luminescence at 543 nm. Excitation was done at 294 nm to excite the DNA and the luminescence peak at 543 nm is only observed in the presence of Tb^3+^ and junction. (**Inset**) Excitation and emission spectra of the Tb luminescence; the ^5^D_4_→^7^F_5_ transition at 543 nm is highlighted.

We directly explored Ln(III) binding to the junction using Tb^3+^ (Figure 4). Using an excitation wavelength of 294 nm to excite the DNA but avoid direct excitation of the Tb^3+^, we found that the Tb^3+^ exhibited a DNA-sensitized emission at 543 nm in the presence of the junction. This emission wavelength corresponded to the ^5^D_4_→^7^F_5_ transition of the Tb^3+^ ion and was only observed in the presence of both ion and junction and not in the absence of either. The luminescence observed under these conditions is consistent with Tb^3+^ binding to the junction. Analysis of the integrated intensity increase at 543 nm associated with increasing concentrations of Tb^3+^ yielded a *K*_d_ of 1.2 µM, which is larger than the *K*_d_ value (≤0.2 µM) determined by FRET (Figure 1). The larger *K*_d_ value based on the Tb^3+^-enhanced luminescence reflects both site-specific and non-specific ion binding to the junction (Supplementary Material, Figure S5). The smaller *K*_d_ value obtained by FRET measurements solely arises from the specific binding of Tb^3+^ to the center of the DNA junction, which affects the distance between the two dyes. These findings suggest that Ln(III) luminescence probes specific and non-specific interactions of the ions with the junction.

**Figure 5:**
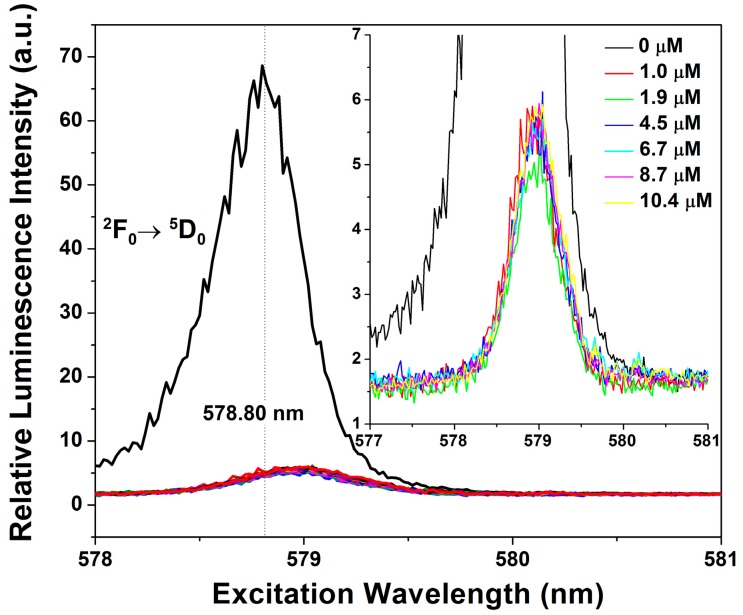
Excitation scan of the Eu^3+^ transition, ^2^F_0_→^5^D_0_, centered at a wavelength of 578.80 nm. The concentration of europium is 10 μM, while junction is titrated in to reach the same concentration. Junction binding quenched the overall europium luminescence and produced a slight red-shift in the excitation peak to 579.00 nm. (**Inset**) Inset highlights the peaks from 1.0 to 10.4 µM Eu^3+^, which are relatively narrow (fwhm = 1 nm) and uniform.

Although quite different in luminescent properties, Eu^3+^ and Tb^3+^ have similar physical characteristics and are expected to interact with the junction in the same manner [31,32]. The weaker luminescence of Eu^3+^ required the use of a pulsed laser source to generate the emission and excitation spectra. Nevertheless, because of the non-degeneracy of the ^7^F_0_→^5^D_0_ luminescence transition of Eu^3+^ a wealth of information can be gained regarding the coordination environment and the geometry of the site. We used Eu^3+^ luminescence to probe the environment of the putative ion-binding site (Supplementary Material, Figure S6). Using the information from the ITC and FRET experiments, we employed junction and ion concentrations that primarily investigated the stronger binding site. As shown in Figure 5, titration of the junction into an aqueous solution of Eu^3+^ led to a dramatic quenching of the emission and a slight shift of the peak emission to longer wavelength. The resulting Eu^3+^–4WJ peak was relatively narrow and symmetric with a full width at half maximum (fwhm) of 1 nm, consistent with a single class of binding site. A similar result is observed if Eu^3+^ is titrated into a solution of the junction; however, at the concentrations used in this titration, both classes of binding are expected to occur (Supplementary Material, Figure S7). The observed binding behavior was comparable to what had previously been reported for Eu^3+^ binding to the hammerhead ribozyme [34,35] and other RNA–Eu^3+^ complexes [36,37]. We cannot rule out the possibility; however, that we were not able to resolve a binding class with a different environment but similar energy levels.

#### Coordination Geometry and Number of Ions Bound

Further support for the observation of a homogenous binding environment under these conditions is obtained from relaxation data of the Eu^3+^ luminescence. For these measurements, we used the ^2^F_0_→^5^D_2_ transition as it generated a more intense emission and was useful for amplifying the signal from the quenched species (Supplementary Material, Figure S4). For Eu^3+^ in aqueous solutions, luminescence primarily occurs out of the ^5^D_0_ state regardless of excitation, as relaxation of the higher energy states is rapidly quenched due to vibronic coupling with OH groups from water. Since the lifetime is governed by the coupling with OH groups, it can be used to determine the number of bound water molecules. This dependence of the Eu^3+^ lifetime on coordination environment results because vibronic energy transfer to O–H vibrations is the dominant relaxation pathway out of the excited state and the rate of relaxation is proportional to the number of coordinating water molecules [32,38]. Thus, we compared the Eu^3+^–4WJ lifetime with that of aqueous Eu^3+^ to provide an indication of the coordination environment. The lifetime data for the Eu^3+^–4WJ complex is mono-exponential and yields an average lifetime of 124.8 µs; the observation of a single lifetime is consistent with the presence of only one binding environment for the stronger affinity site. The average measured lifetime of aqueous europium was found to be 111.9 ± 0.4 μs using the lifetimes obtained with 464.1 and 464.5 nm excitation (Table 2), which agrees well with previously determined values [39]. The anomalously long lifetime observed with 465 nm excitation is attributed to impurities in the solution.

To determine the number of coordinating waters, we used the following relationship derived by Kimura and coworkers [38]

(1) $$q=A\prime \left(\frac{1}{\tau_{H_{2}O}}-\alpha \prime \right)$$

where *q* is the number of coordinating waters, *A*’ is 1.11 ms, α’ is 0.44 ms^−1^ and $\tau_{H_{2}O}$ is the lifetime in water. The *A*’ and α’ constants are empirically-derived from water/non-aqueous solvent mixtures assuming that other deactivation pathways are not present.

We obtained a *q* value of 8.4 ± 0.2 from this relationship, indicating that on average the inner sphere of these europium ions contained eight water molecules when bound to the junction, compared to nine water molecules coordinated to Eu^3+^ in aqueous solution. This finding suggested that the bound ion loses one H_2_O to directly coordinate to the DNA. This direct coordination to the DNA is in keeping with the exothermic binding reaction observed by ITC. In previous work, Eu^3+^ was found to bind to an RNA GAAA tetraloop, with a similar slight dehydration of the ion (removal of one coordinated water molecule) upon binding [36,37].

**Figure 6:**
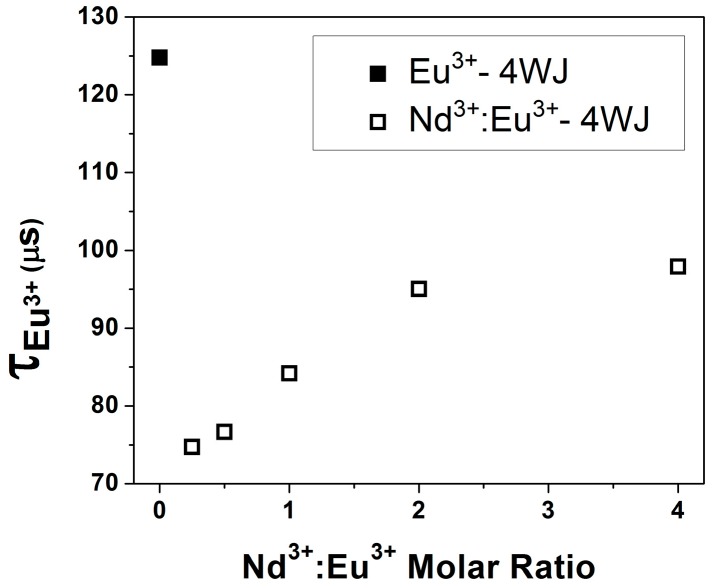
The addition of Nd^3+^ quenched Eu^3+^–4WJ luminescence because of energy transfer. Increasing concentrations of Nd^3+^ displaced Eu^3+^ from the junction leading to a longer lifetime that approached the Eu^3+^ lifetime observed in the absence of junction and Nd^3+^ (111.9 ± 0.4 μs). The Eu^3+^ concentration was 64 μM, while the concentration of junction was 6.3 μM. Other conditions were as described in Figure 5.

We used luminescence resonance energy transfer (LRET) to measure the relative proximity of the bound Ln(III) ions. As previously shown for protein bound Ln(III) ions, this non-radiative resonance energy transfer can be described by a Förster mechanism and is effective for determining inter-metal ion distances [32]. For the 4WJ, Eu^3+^ is used as the energy donor and the Nd^3+^ was titrated into the solution as the acceptor (Figure 6). Previous results have shown that Nd^3+^ is a very efficient acceptor of Eu^3+^ luminescence. In the current work, the addition of Nd^3+^ to the solution led to an immediate quenching of the Eu^3+^ luminescence from approximately 125 µs in the absence of Nd^3+^ to 74.7 µs at a Nd^3+^:Eu^3+^ ratio of 0.25. As the concentration of Nd^3+^ was increased, the lifetime increased and asymptotically approached the lifetime of aqueous Eu^3+^. This lengthening of the lifetime was consistent with displacement of Eu^3+^ from the binding site by Nd^3+^. Despite the presence of many Eu(III) species (Eu^3+^:4WJ, Eu^3+^Nd^3+^:4WJ and Eu^3+^(aq)) in the solution we were not able to resolve multiple species from the luminescence decays and attribute this to the fact that the Eu^3+^ luminescence is considerably quenched when Eu^3+^ is associated with the junction relative to the free ion in water (see Figure 5). We note that the only species that would exhibit any quenching from LRET is Eu^3+^Nd^3+^:4WJ. We estimated the degree of energy transfer from the first point of the titration to the point at which the molar ratio of Nd^3+^:Eu^3+^ was 1:1. The transfer efficiency ranged from 0.4 to 0.325 giving an estimated distance range between the two ions from 6.4 to 6.8 Å. We used an *R*_0_ value of 6.0 Å, measured previously for rGGCC complexes to calculate these distances [40]. These values may represent an upper limit for the inter-ion distance as the lifetimes of Eu(III) bound to the junction (125 µs) and free in solution (110 µs), also contribute to the observed lifetime. Despite these contributions, the distance obtained is in good agreement with the molecular dynamics (MD) simulations of Wheatley *et al.* [23], who detected two regions of high ion density that were 7 Å apart in a homologous junction. In a crystal structure of a junction, two ions were observed in the center separated by a distance of 12 Å [21]. A strict comparison is not possible as the crystallography and MD studies used different ions with smaller ionic radii relative to the trivalent lanthanides. Since the concentrations of Nd^3+^ and Eu^3+^ were higher than the junction, there could have been interaction with more than one donor or acceptor; however, given the relative proximity needed for efficient energy transfer, the relatively short residence times of non-specific interactions and the fractional occupancy of the ion atmosphere we expect this effect to be negligible. The overall consistency of our lanthanide ion results with those obtained with Mg^2+^ argues that we monitored similar binding events and investigated the same binding interaction. These results in combination with the ITC results strongly support a model in which site-specific binding of two ions in relative proximity to each other is needed for junction folding.

## 3. Experimental Section

### 3.1. Oligonucleotide Preparation and Labeling

Single-stranded DNA oligonucleotides for the J3 junction were purchased from Integrated DNA Technologies (Coralville, IA, USA): **R**, CCT TCA ACC ACC GCT CAA CTC AAC TGC AGT CTG G; **X**, CCA GAC TGC AGT TGA GTC CTT GCT AGG ACG GAG G; **B**, CCT CCG TCC TAG CAA GGG GCT GCT ACC GGA AGG G; and **H**, CCC TTC CGG TAG CAG CCT GAG CGG TGG TTG AAG G. Strands were purchased (IDT) either HPLC-purified or in crude form and purified in the gel. Gel purification was accomplished as previously described [16] using a denaturing-gel containing 15% polyacrylamide (38:2) with 7 M urea and TBE (22.5 mM Tris base, 22.5 mM boric acid, and 0.625 mM Na_2_EDTA at pH 8.3). DNA bands were detected through UV shadowing and cut from the gel; the DNA was recovered by electroelution (Schleicher and Schuell Elutrap, Concord, NH, USA) and dialyzed against 12 L of dH_2_O. For dye conjugation, a C6 amino linker was attached to the 5′-end. The dyes, 5-carboxytetramethylrhodamine SE (TAMRA) and 5-carboxyfluorescein SE (FAM) were obtained from Molecular Probes (Thermo Fisher Scientific, Waltham, MA, USA) and attached to the strands following their procedures [41]. Labeled strands were ethanol-precipitated to remove the excess dye. A correction factor particular to each dye was used to ensure accurate concentration determination of dye-conjugated oligonucleotides.

### 3.2. Junction Construction

Four way junctions were successfully formed in a solution of 300 mM NaCl, and 10 mM Tris–HCl pH 7.4 with each of the strands at equimolar concentrations. Samples were kept at 68 °C for two hours and cooled slowly to room temperature (6–8 h). Junction formation in annealed samples was routinely confirmed through observation on a 6.5% acrylamide native gel. Insufficiently annealed samples (<90% by densitometry) were purified as described for single strand but using a native gel at 4 °C. Dye-labeled junction samples were prepared in the same way.

### 3.3. FRET Measurements and Analysis

FRET measurements were recorded with a FluoroMax-2 instrument (Horiba Jobin-Yvon, Edison, NJ, USA). Experimental conditions were 10 °C with a constant stirring rate of 300 rpm using 50 nM of 4WJ DNA. Spectra were obtained on doubly labeled junctions as well as donor only and acceptor only junctions using 3 mm × 3 mm siliconized glass cuvettes (Starna Cell, Inc., Atascadero, CA, USA). For the donor, FAM, samples were excited at 494 nm and emission spectra were recorded between 508 and 652 nm. For the acceptor, TAMRA, an excitation wavelength of 555 nm was used and emission spectra were measured from 570 to 700 nm. Scans were obtained at a resolution of 2 nm/pt and a speed of 1s/pt with excitation and emission slits at 4 and 8 nm bandpass, respectively. All spectra were obtained under magic angle conditions (excitation polarizer = 0° and emission polarizer = 55°).

The quantity (ratio)_A_, which compares the increase of acceptor fluorescence emission upon exciting the donor relative to direct excitation of the acceptor on the labeled junction, was used to calculate the FRET efficiencies [14,42].

(2) $${\left(\text{ratio}\right)}_{A}=\frac{F_{\text{AD}}\left(\lambda_{A}^{\text{em}}\right)}{F_{A}\left(\lambda_{A}^{\text{em}}\right)}$$

(3) $$E=\left(\frac{F_{\text{AD}}\left(\lambda_{A}^{\text{em}}\right)}{F_{A}\left(\lambda_{A}^{\text{em}}\right)}-\frac{\varepsilon_{A}\left(494\right)}{\varepsilon_{A}\left(555\right)}\right)\left(\frac{1}{f_{D}}\right)\left(\frac{\varepsilon_{A}\left(555\right)}{\varepsilon_{D}\left(494\right)}\right)$$

where *F*_AD_ is the acceptor emission of the doubly-labeled molecule obtained at the donor excitation wavelength, *F*_A_ is the acceptor emission of the doubly-labeled molecule observed by direct excitation of the acceptor, ε_A_ is the acceptor extinction coefficient (ε_A,555_ = 89,100 M^−1^·cm^−1^) and ε_D_ is the donor extinction coefficient, FAM (ε_D,494_ = 78,000 M^−1^·cm^−1^), and 1/*f*_D_ is the fraction of donor-labeled molecules. Extinction coefficients were taken from the dye manufacturer (Molecular Probes, Thermo Fisher Scientific, Waltham, MA, USA). Ratio_A_ was normalized to the fluorescence of a donor-only sample at each titration point as previously described [43]. Consequently, the efficiency values obtained were corrected for any donor photobleaching and any ion-induced quenching of FAM fluorescence. To determine the relative quantum yields we compared the fluorescence from the singly-labeled donor 4WJ to fluorescein in 0.1 M NaOH. The overlap integral was calculated from singly-labeled FAM and TAMRA junctions as previously described [16]. The *R*_0_ values were determined at the initial and final points of the titration to account for any ion-induced changes in the quantum yield and were 50 ± 2 Å. The angle between adjacent junction arms was calculated using the following equation:

(4) $$c^{2}=a^{2}+b^{2}-2ab\cos\theta$$

where *a* and *b* are the length of the arms, assumed to be the same, 17 bp on each side. In this calculation, the arms were assumed to be straight with no bending or kinking of the helix. The junction angle is opposite side c, which is the FRET-measured distance between donor and acceptor [16].

FRET efficiency (*E*) as a function of ion concentration [M^+^] was fit to a two-state model for the folding of the junction:

(5) $$E=E_{0}+\frac{K_{a}{\left[M^{+}\right]}^{n}\left(E_{i}-E_{0}\right)}{1+K_{a}{\left[M^{+}\right]}^{n}}$$

where *E*_0_ is the initial value, *E_i_* is the final value, *K*_a_ is the apparent association constant and *n* is the Hill coefficient, typically 1–1.1 indicative of non-cooperative binding; all of these parameters were allowed to vary in the fitting of the data (OriginLab Corp., Northampton, MA, USA) [14,19].

### 3.4. Isothermal Titration Calorimetry

ITC experiments were performed on a Microcal VP-ITC instrument, following the standard protocol given in the MicroCal VP-ITC manual. (MicroCal 2002) Experiments were conducted in 0.5 mM NaCl, 10 mM Tris–HCl pH 7.4 buffer with Mg^2+^ as the titrant and junctions in the sample chamber. Titrations were performed at 10 °C with a syringe stir rate of 307 rpm. When further titration was desired upon completion of the first round, the syringe was reloaded with titrant and titration continued. ConCat32 (software available from MicroCal) was used for concatenation of the results. Normalized changes in enthalpy (NdH) were calculated through Origin 7.0, and the corresponding values of NdH were corrected for any enthalpy changes occurring in the absence of junction. Using Origin 7.0, data were fit assuming either one or two sets of sites in the macromolecule [44].

The cumulative heat absorbed or released as a consequence of ions binding is given by the sum of the heats of binding to each class of site.

(6) $$Q=V\sum_{i}\Delta H_{i}\left[L_{B,i}\right]$$

where *V* is the reaction volume, ∆*H* is the enthalpy of binding (mol·ion)^−1^ and *L*_B_ is the concentration of bound ion. This can be re-expressed as:

(7) $$Q=V\left[M\right]\sum_{i}\frac{n_{i}\Delta H_{i}K_{i}\left[L\right]}{1+K_{i}\left[L\right]}$$

where [*M*] is the total concentration of 4WJ available for binding ions, *K_i_* is the site association constant for each class of binding site, *n_i_* is the number of binding sites of each class on each junction and [*L*] is the concentration of free ligand. Analysis of the data is done in terms of the individual heat associated with each injection to minimize the propagation of errors. The closed form of the equation used for the two classes of independent binding sites for the 4WJ (Equation (8)) is solved numerically.

(8) $$Q=V\left[M\right]\left(\frac{n_{1}\Delta H_{1}K_{1}\left[L\right]}{1+K_{1}\left[L\right]}+\frac{n_{2}\Delta H_{2}K_{2}\left[L\right]}{1+K_{2}\left[L\right]}\right)$$

The fits with two classes of binding sites were performed using the van't Hoff determined parameters for ∆*H*_1_ and *K*_1_ (described below). These independently determined parameters were kept constant, while *n*_1_, *n*_2_, ∆*H*_2_, and *K*_2_ were allowed to vary. ∆*S* is calculated from the fitted parameters.

The thermodynamic parameters for ion-induced conformational changes were determined using the van’t Hoff relationship [45]

(9) $$\Delta H_{\text{HV}}=-R\left[\frac{d\ln K_{a}}{d\left(1/T\right)}\right]$$

where *K*_a_ values were determined from Mg^2+^ titrations as measured by FRET performed at 4, 10, 20, 25, and 30 °C.

### 3.5. Lanthanide Luminescence

Excitation spectra of the terbium-bound junctions were obtained with emission at 543 nm. An excitation wavelength of 294 nm was used for emission spectra. Slits were set to 2 nm bandpass with an integration time of 1 s and measurements were conducted in a pH 7.4 MES buffer with 0.5 mM NaCl. Analysis of terbium binding and calculation of *K*_d_ values were performed as described for FRET data.

Europium(III) chloride solutions were standardized by complexometric titration of Arsenazo Indicator in complexation with EDTA [46]. Europium excitation spectra and luminescence lifetime measurements were made using a spectroscopic system powered by an Nd:YAG laser coupled with a master oscillator power oscillator (MOPO) (Department of Chemistry, University at Buffalo, State University of New York, Buffalo, New York). The pump laser was a Nd:YAG laser (Spectra-Physics, model Quanta Ray PRO-270-10, Santa Clara, CA, USA) operating at 10 Hz with energies of 550 mJ/pulse and linewidths of 0.003 cm^−1^ at 355 nm. These pulses seeded the MOPO, or master oscillator power oscillator, (Spectra-Physics, model Quanta Ray MOPO-SL, Santa Clara, CA, USA). The MOPO outputs (signal, idler) are continuously tunable from 420–690 nm (signal) and 735–1800 nm (idler) with a line width of 0.2 cm^−1^. The desired excitation wavelengths are obtained from the MOPO using a general purpose interface bus (GPIB) associated with a personal computer. The pulse duration from the MOPO is 10–12 ns. The average pulse energy is 57 ± 8 mJ between 450 and 590 nm [39]. Binding measurements were conducted with junction samples at concentrations between 8–10 µM in a 0.5 mM NaCl, 10 mM Tris–HCl pH 7.4 buffer. Europium ion was either titrated into an 8.8 µM junction solution or the junction was titrated into a 10 µM Eu^3+^ solution.

## 4. Conclusions

In summary, we have used a number of different methods to examine the ion binding sites in a DNA 4WJ. These results collectively support a model in which ion binding to the junction is largely mediated by electrostatic interactions, where multivalent ions bind with higher affinity than monovalent ions. Isothermal titration experiments, FRET and lanthanide luminescence results were indicative of two classes of ion binding sites. Ion binding to the higher affinity site led to folding of the junction into a stacked conformation. This binding was largely enthalpically driven and the stoichiometry of binding was approximately 2. The second class of site was weaker and the binding was largely entropically driven consistent with displacement of water and other ions upon binding. We suggest that this binding is associated with charge neutralization of the phosphate backbone where either the Ln(III) ions or the Mg^2+^ ions are displacing some of the condensed counterions. Lanthanide luminescence pointed to a relatively homogeneous ion environment and further suggested that the ions were only slightly dehydrated upon binding, making only one inner sphere contact with the DNA. LRET measurements further indicated that the ions are located within 6–7 Å of each other. These results support a model in which site-specific binding of two ions to the center of the junction is required for the folding of 4WJ into a stacked conformation.

## Figures and Tables

**Table 1:** Thermodynamic Parameters of Ion Binding.

Two Class Model ^a^	Class 1	Class 2
Stoichiometry	2.1 ± 0.4	15.9 ± 2.0
*K*_a_ (M^−1^)	40,180 ± 13,710 ^b^	25,000 ± 3000
∆*H* (kcal/mol)	−14.9 ± 0.6 ^b^	1.9 ± 0.5
∆*S* (cal/(°·mol))	−31.5 ± 0.2 ^b^	26.8 ± 1.2

^a^ The ITC data shown in Figure 2 required two classes of binding site to describe both the exothermic and endothermic components. The χ^2^ parameter for this fit was 385 compared to 5216 for a single class of binding site. Single component fits to the data are shown in Figure S3 (Supplementary Material); ^b^ Determined from FRET experiments at different temperatures (Supplementary Material, Figure S4) and a van’t Hoff analysis (see Figure 3) as described in the text.

**Table 2:** Eu^3+^ lifetime data and estimate of coordinating water molecules.

Excitation Wavelength (nm)	Eu^3+^ (aq)		Eu^3+^–4WJ	
	$\tau_{H_{2}O}$ (μs)	*q*	$\tau_{H_{2}O}$ (μs)	*q*
464.1	105.9	9.9	121.4	8.6
464.5	117.8	8.9	124.4	8.4
465.0	140.0	7.5	128.7	8.2
Average	111.9 ± 8.4 ^a^	9.4 ± 1.2	124.8 ± 3.7	8.4 ± 0.2

^a^ Average of data collected from excitation wavelengths 464.1 and 464.5 nm only.

## Supplementary Materials

Supplementary materials can be found at http://www.mdpi.com/1422-0067/17/3/366/s1.

## References

[1] Brazda V., Laister R.C., Jagelska E.B., Arrowsmith C. (2011). Cruciform structures are a common DNA feature important for regulating biological processes. BMC Mol. Biol..

[2] Lu S., Wang G., Bacolla A., Zhao J., Spitser S., Vasquez K.M. (2015). Short inverted repeats are hotspots for genetic instability: Relevance to cancer genomes. Cell Rep..

[3] Khuu P.A., Voth A.R., Hays F.A., Ho P.S. (2006). The stacked-x DNA Holliday junction and protein recognition. J. Mol. Recognit..

[4] Jones M.R., Seeman N.C., Mirkin C.A. (2015). Nanomaterials. Programmable materials and the nature of the DNA bond. Science.

[5] Duckett D.R., Murchie A.I., Diekmann S., von Kitzing E., Kemper B., Lilley D.M. (1988). The structure of the Holliday junction, and its resolution. Cell.

[6] Churchill M.E., Tullius T.D., Kallenbach N.R., Seeman N.C. (1988). A Holliday recombination intermediate is twofold symmetric. Proc. Natl. Acad. Sci. USA.

[7] Cooper J.P., Hagerman P.J. (1989). Geometry of a branched DNA structure in solution. Proc. Natl. Acad. Sci. USA.

[8] Miick S.M., Fee R.S., Millar D.P., Chazin W.J. (1997). Crossover isomer bias is the primary sequence-dependent property of immobilized Holliday junctions. Proc. Natl. Acad. Sci. USA.

[9] Declais A.C., Lilley D.M. (2008). New insight into the recognition of branched DNA structure by junction-resolving enzymes. Curr. Opin. Struct. Biol..

[10] Hays F.A., Watson J., Ho P.S. (2003). Caution! DNA crossing: Crystal structures of Holliday junctions. J. Biol. Chem..

[11] Eichman B.F., Ortiz-Lombardia M., Aymami J., Coll M., Ho P.S. (2002). The inherent properties of DNA four-way junctions: Comparing the crystal structures of Holliday junctions. J. Mol. Biol..

[12] Ho P.S., Eichman B.F. (2001). The crystal structures of DNA Holliday junctions. Curr. Opin. Struct. Biol..

[13] Ortiz-Lombardia M., Gonzalez A., Eritja R., Aymami J., Azorin F., Coll M. (1999). Crystal structure of a DNA Holliday junction. Nat. Struct. Biol..

[14] Clegg R.M., Murchie A.I., Zechel A., Carlberg C., Diekmann S., Lilley D.M. (1992). Fluorescence resonance energy transfer analysis of the structure of the four-way DNA junction. Biochemistry.

[15] Murchie A.I., Clegg R.M., von Kitzing E., Duckett D.R., Diekmann S., Lilley D.M. (1989). Fluorescence energy transfer shows that the four-way DNA junction is a right-handed cross of antiparallel molecules. Nature.

[16] Vitoc C.I., Mukerji I. (2011). HU binding to a DNA four-way junction probed by Förster resonance energy transfer. Biochemistry.

[17] Joo C., McKinney S.A., Lilley D.M.J., Ha T. (2004). Exploring rare conformational species and ionic effects in DNA Holliday junctions using single-molecule spectroscopy. J. Mol. Biol..

[18] McKinney S.A., Declais A.C., Lilley D.M., Ha T. (2003). Structural dynamics of individual Holliday junctions. Nat. Struct. Biol..

[19] Duckett D.R., Murchie A.I., Lilley D.M. (1990). The role of metal ions in the conformation of the four-way DNA junction. EMBO J..

[20] Mollegaard N.E., Murchie A.I., Lilley D.M., Nielsen P.E. (1994). Uranyl photoprobing of a four-way DNA junction: Evidence for specific metal ion binding. EMBO J..

[21] Thorpe J.H., Gale B.C., Teixeira S.C., Cardin C.J. (2003). Conformational and hydration effects of site-selective sodium, calcium and strontium ion binding to the DNA Holliday junction structure d(TCGGTACCGA)(4). J. Mol. Biol..

[22] Hyeon C., Lee J., Yoon J., Hohng S., Thirumalai D. (2012). Hidden complexity in the isomerization dynamics of Holliday junctions. Nat. Chem..

[23] Wheatley E.G., Pieniazek S.N., Mukerji I., Beveridge D.L. (2012). Molecular dynamics of a DNA Holliday junction: The inverted repeat sequence d(CCGGTACCGG)(4). Biophys. J..

[24] Watson J., Hays F.A., Ho P.S. (2004). Definitions and analysis of DNA Holliday junction geometry. Nucleic Acids Res..

[25] Auclair S.M., Oliver D.B., Mukerji I. (2013). Defining the solution state dimer structure of *Escherichia coli* SecA using Förster resonance energy transfer. Biochemistry.

[26] Ohtaki H., Radnai T. (1993). Structure and dynamics of hydrated ions. Chem. Rev..

[27] Lipfert J., Doniach S., Das R., Herschlag D. (2014). Understanding nucleic acid-ion interactions. Annu. Rev. Biochem..

[28] Jen-Jacobson L., Engler L.E., Jacobson L.A. (2000). Structural and thermodynamic strategies for site-specific DNA binding proteins. Structure.

[29] Manning G.S. (1978). The molecular theory of polyelectrolyte solutions with applications to the electrostatic properties of polynucleotides. Q. Rev. Biophys..

[30] Matulis D., Rouzina I., Bloomfield V.A. (2000). Thermodynamics of DNA binding and condensation: Isothermal titration calorimetry and electrostatic mechanism. J. Mol. Biol..

[31] Morrow J.R., Andolina C.M., Sigel A., Sigel H., Sigel R.K.O. (2012). Spectroscopic investigations of lanthanide ion binding to nucleic acids. Interplay between Metal Ions and Nucleic Acids.

[32] Horrocks W.D., Riordan J.F., Vallee B.L. (1993). Luminescence spectroscopy. Metallobiochemistry Part c: Spectroscopic and Physical Methods for Probing Metal Ion Environments in Metalloenzymes and Metalloproteins.

[33] Pechlaner M., Sigel R.K.O., Sigel A., Sigel H., Sigel R.K.O. (2012). Characterization of metal ion-nucleic acid interactions in solution. Interplay between Metal Ions and Nucleic Acids.

[34] Feig A.L., Panek M., Horrocks W.D., Uhlenbeck O.C. (1999). Probing the binding of Tb(III) and Eu(III) to the hammerhead ribozyme using luminescence spectroscopy. Chem. Biol..

[35] Feig A.L., Scott W.G., Uhlenbeck O.C. (1998). Inhibition of the hammerhead ribozyme cleavage reaction by site-specific binding of Tb. Science.

[36] Escudier J.M., Dupouy C., Fountain M.A., del Mundo I.M., Jacklin E.M., Morrow J.R. (2009). Synthesis and luminescence properties of a trinucleotide-europium(III) complex conjugate. Org. Biomol. Chem..

[37] Mathews R.A., Rossiter C.S., Morrow J.R., Richard J.P. (2007). A minimalist approach to understanding the efficiency of mononuclear Zn(II) complexes as catalysts of cleavage of an RNA analog. Dalton Trans..

[38] Kimura T., Nagaishi R., Kato Y., Yoshida Z. (2001). Luminescence study on preferential solvation of Europium(III) in water/non-aqueous solvent mixtures. J. Alloy. Compd..

[39] Andolina C.M., Holthoff W.G., Page P.M., Mathews R.A., Morrow J.R., Bright F.V. (2009). Spectroscopic system for direct lanthanide photoluminescence spectroscopy with nanomolar detection limits. Appl. Spectrosc..

[40] Andolina C.M., Morrow J.R. (2011). Luminescence resonance energy transfer in heterodinuclear LnIII complexes for sensing biologically relevant anions. Eur. J. Inorg. Chem..

[41] Thermo Fisher Scientific Amine Reactive Probe Labeling Protocol. https://www.thermofisher.com/us/en/home/references/protocols/cell-and-tissue-analysis/labeling-chemistry-protocols/amine-reactive-probe-labeling-protocol.html.

[42] Lakowicz J.R. (2006). Principles of Fluorescence Spectroscopy.

[43] Clegg R.M., Murchie A.I., Lilley D.M. (1994). The solution structure of the four-way DNA junction at low-salt conditions: A fluorescence resonance energy transfer analysis. Biophys. J..

[44] Freire E., Mayorga O.L., Straume M. (1990). Isothermal titration. Anal. Chem..

[45] Marky L.A., Breslauer K.J. (1987). Calculating thermodynamic data for transitions of any molecularity from equilibrium melting curves. Biopolymers.

[46] Lyle S.J., Rahman M.M. (1963). Complexometric titration of yttrium and the lanthanons. I. A comparison of direct methods. Talanta.

