# Supplementary material for: Probing the Ion Binding Site in a DNA Holliday Junction Using Förster Resonance Energy Transfer (FRET)
Source: Int J Mol Sci. 2016 Mar 10;17(3):366. doi: 10.3390/ijms17030366 (PMC4813226; doi:10.3390/ijms17030366)
Supplement: Supplementary file 1 [file ijms-17-00366-s001.pdf]

Supplementary Materials: Probing the Ion Binding Site in a DNA Holliday Junction Using Förster Resonance Energy Transfer (FRET)

Jacob L. Litke, Yan Li, Laura M. Nocka and Ishita Mukerji

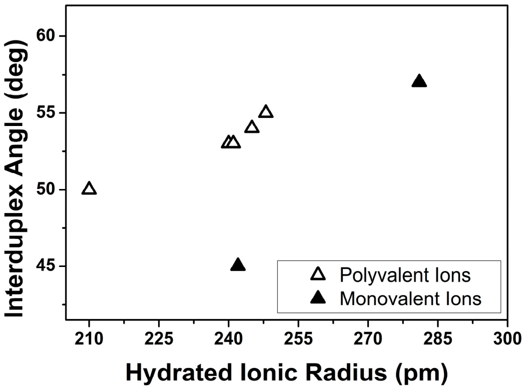

(**a**)

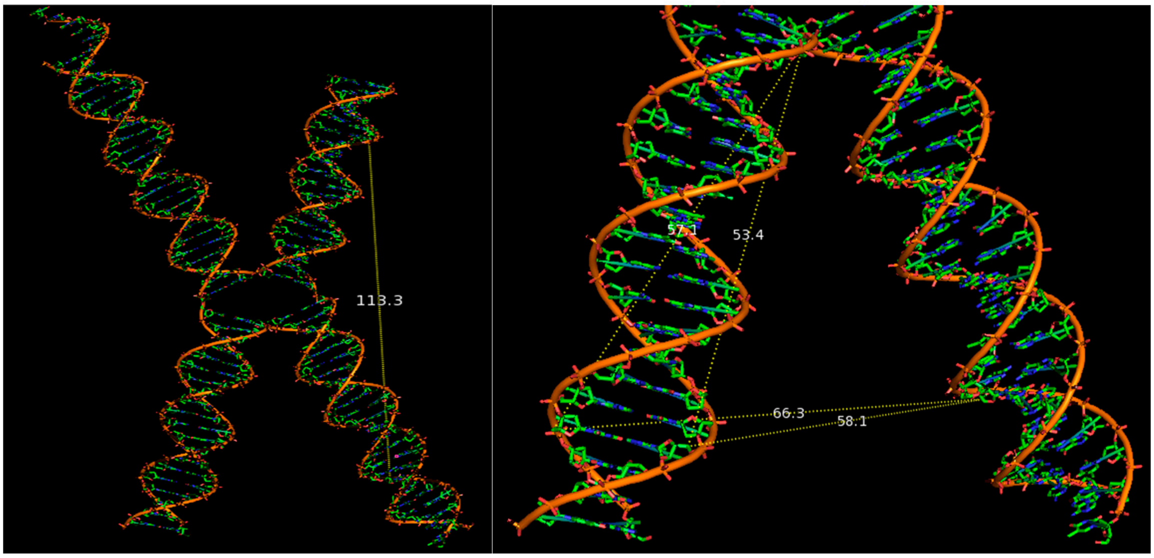

(**b**)

Figure S1. (**a**) Interduplex angle determined from FRET measured distances as described in the text. In general we observed for both monovalent and polyvalent ions that the interduplex angle was proportional to the hydrated ion radius. The experimentally determined distances and associated interduplex angles with errors are given in Table S1; (**b**) Model of a stacked Holliday junction with an IDA of 45°. On the left the distance between the C5′ atoms of the 17th bp from the junction center of two co-axially stacked arms is used to estimate the distance between dyes in the isoI conformation (113 Å). Right: Distances are shown from the top of the arm to the 17th bp on both strands (53 and 57 Å). Also shown are the distances from the 17th bp of one arm to the 17th bp of the adjacent arm (58 and 66 Å) to estimate the effect of helix position on the distances determined. In both cases, we find that the difference in distance introduced by the position on the helix is smaller than the error in the distance reported from the relative mobility of the dyes. All distances are measured at the C5′ atom.

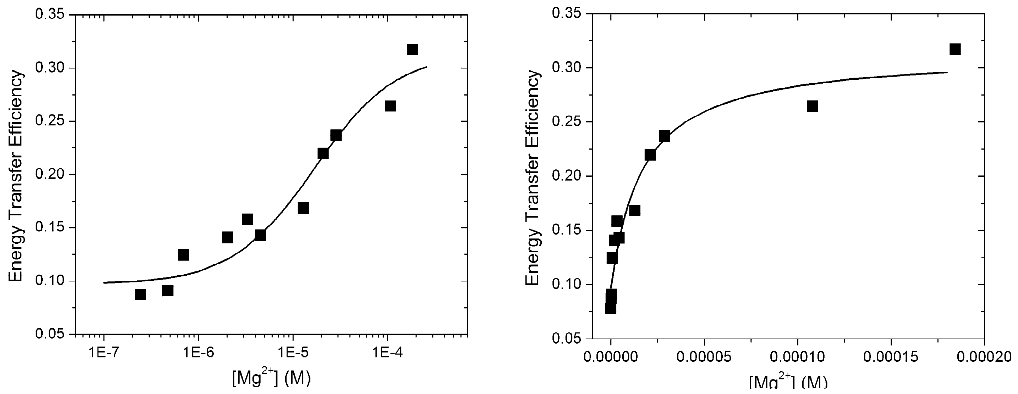

Figure S2. Analysis of Mg^{2+}:4WJ binding interaction using a model of two identical, non-interacting binding sites ($\alpha = n \times k[Mg^{2+}]/(1 + k[Mg^{2+}])$ where α = fraction folded, n is the number of binding sites and k is the microscopic association constant). Data shown are taken from Figure 1B in the manuscript. This fitting model gave comparable K_a values to the apparent K_a values obtained from fitting the data to Equation (5) in the manuscript (59,400 M^{-1} *vs.* 45,400 M^{-1}). We note that in order to obtain the two-site binding fit, the number of binding sites needed to be constrained to 2. (**Left**): Data and fit are shown plotted on a log scale; (**Right**): Data and fit are shown plotted on a linear scale.

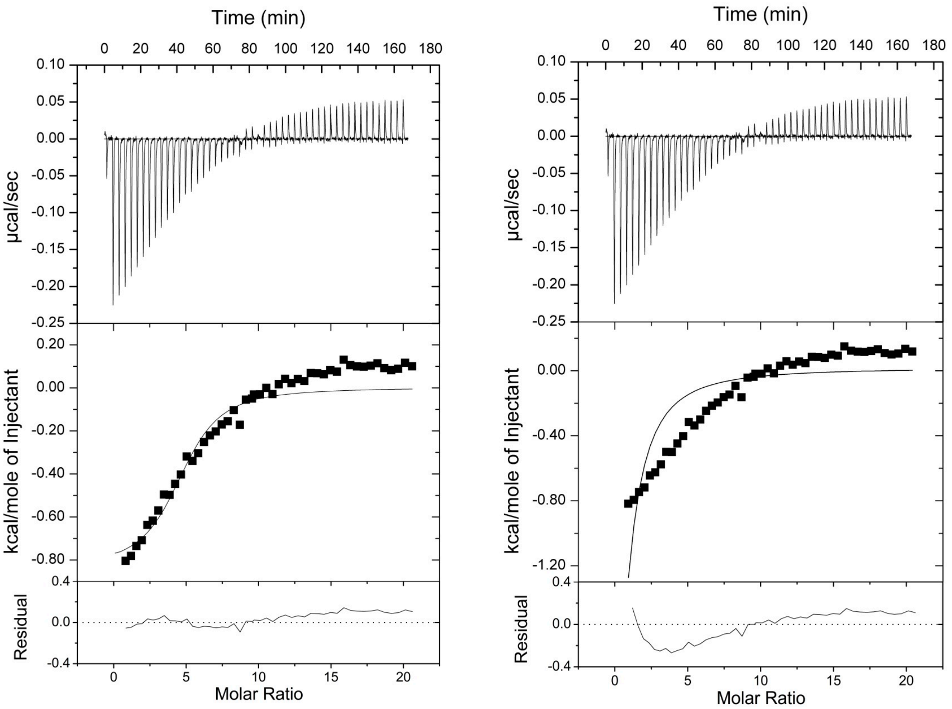

(**a**) (**b**)

Figure S3. Representative fits of the ITC data to one binding component. (**a**) Fitting of the ITC data to a one-to-one binding model where all parameters were allowed to vary; (**b**) Fitting of the ITC data to a one-to-one binding model, where K_a and ΔH were held fixed to the van't Hoff determined parameters of 40,181 M^{-1} and −14.9 kcal/mol, respectively. Residuals depicted are generated between the actual data and the fit shown. All parameters of the fits are given in Table S2. Generally, χ^2-values and visual inspection of the fit and the residuals indicated that another component was needed to adequately describe the data.

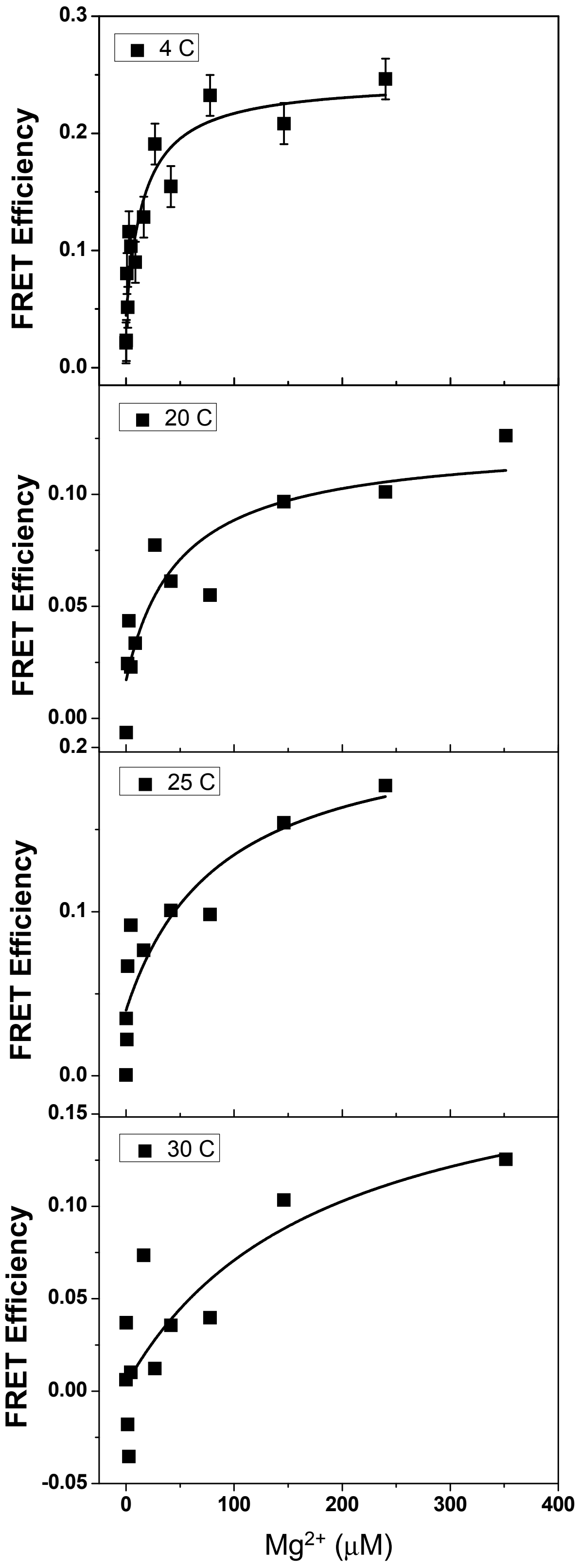

Figure S4. Energy transfer efficiencies measured for the Mg^{2+}-junction interaction measured at 4, 20, 25, and 30 °C. The K_a values were determined as described in the text and used to determine ΔH and ΔS values for the ion binding reaction. All titrations were carried out in a 0.5 mM Na^+ and 10 mM Tris–HCl, pH 7.4 buffer.

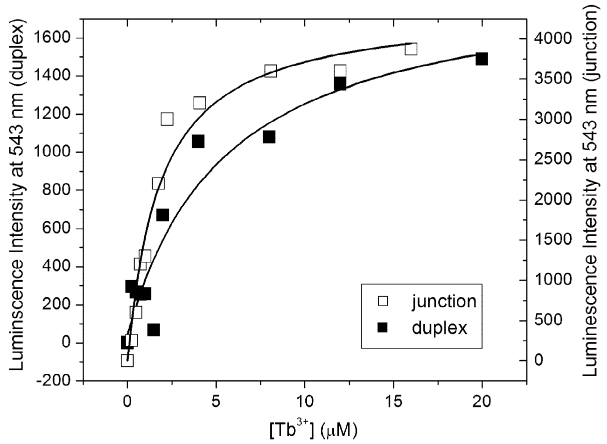

Figure S5. Tb^{3+} binding to 4WJ DNA and 34 bp duplex DNA measured by DNA-sensitized emission at 543 nm. Experimental parameters are the same as for Figure 4. Both data sets were well described by a simple two-state model for ion binding (Equation (5)). The ion binding affinity to the duplex is weaker than ion binding affinity to the junction. However, it is likely that some non-specific ion binding to the junction arms is reflected in the curve shown above and in Figure 4.

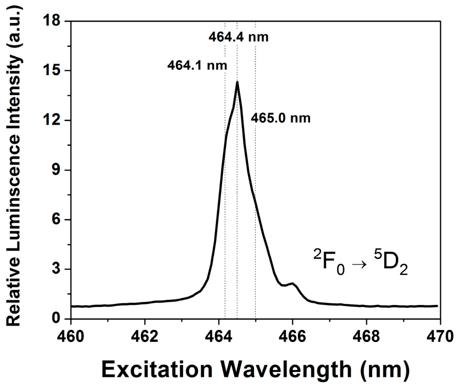

Figure S6. Excitation curve of the $^2F_0 \rightarrow ^5D_2$ transition of aqueous Eu^{3+} at 64 μM, with 6.3 μM junction. Lifetime measurements were performed at the peak wavelength (464.4 nm), and also at two other wavelengths (464.1 and 465.0 nm). Use of this transition allowed for sufficient intensity for lifetime measurements.

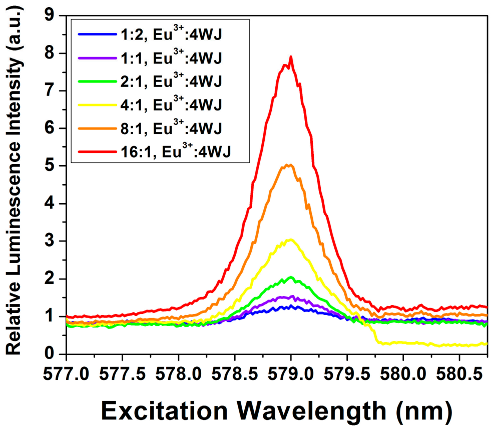

Figure S7. Excitation spectrum of Eu^{3+} luminescence. Titration of Eu^{3+} into a solution of 8.8 μM junction verifies a junction-bound peak at 579.00 nm. No further changes in Eu^{3+} luminescence were observed after a ratio of 16:1, Eu^{3+}:4WJ was reached.

Table S1. FRET efficiencies and calculated junction angles for different ions.

Ion	K_d (app) [a]	R (Å) [b]	IDA (°) [b]	Ion Radius (pm) [c]
Minimal ion concentration	–	80 ± 15	83 ± 20	–
Na^+	29 ± 4 mM [a]	48 ± 9	45 ± 10	242
K^+	5 ± 1 mM [a]	59 ± 11	57 ± 12	281
Mg^{2+}	22 ± 8 μM	53 ± 10	50 ± 11	210
Ca^{2+}	55 ± 8 μM	56 ± 11	53 ± 12	240
Eu^{3+}	3 ± 2 μM	57 ± 11	54 ± 12	245
Tb^{3+}	≤1.2 μM	56 ± 11	53 ± 12	241
Nd^{3+}	≤1.0 μM	58 ± 11	55 ± 12	248

[a] The K_d (app) values were taken from Vitoc and Mukerji [16]. The other K_d (app) values were determined from the data shown in Figure 1; [b] Distances and interduplex angles were determined as described in the text. The calculated error on the distances included the experimental errors in measuring the efficiencies and consideration of the minimal and maximal values of κ^2 based on anisotropy measurements as described previously [25]; [c] Hydrated ion radii were taken from Ohtaki and Radnai [26].

Table S2. Representative fits of the ITC data with one and two components.

	One Class of Binding Site [a]	One Class of Binding Site [b]	One Class of Binding Site [c]	Two Classes of Binding Sites [b]	Two Classes of Binding Sites [a]	Two Classes of Binding Sites [c]
χ^2	5216	22,000	11,000	385	378	378
n (sites)	4.9 ± 0.3	0.27 ± 0.02	1	2.1 ± 0.4	18.5 ± 3.6	1
K_a (M^{-1})	(1.4 ± 0.5) × 10^5	40,181	19,000 ± 3000	40,181	160,000 ± 340,000	3700 ± 7600
ΔH (kcal/mol)	−0.8 ± 0.1	−14.9	−5.4 ± 0.5	−14.9	0.25 ± 0.05	26.1 ± 13.3
ΔS (cal/(°·mol))	20.7	−31.5	0.4	−31.5	24.7	109
n_2 (sites)				15.9 ± 2.0	4.4 ± 0.4	8.6 ± 5.0
$K_{a,2}$ (M^{-1})				(2.5 ± 0.3) × 10^4	(1.1 ± 2.1) × 10^6	(1.4 ± 0.4) × 10^4
ΔH_2 (kcal/mol)				1.9 ± 0.5	−1.6 ± 0.2	−2.2 ± 1.6
ΔS_2 (cal/(°·mol))				26.8	21.9	11.3

[a] All fitting parameters (n, K_a, ΔH) were allowed to vary freely in the fit; [b] In these fits, the parameters $K_{a,1}$ and ΔH_1 were held fixed to parameters independently determined from a van't Hoff analysis of temperature dependent FRET data as described in the text. All other fitting parameters (n_1, n_2, $K_{a,2}$, ΔH_2) were allowed to vary freely; [c] In these fits the n_1 parameter was held fixed at 1, all other parameters (n_2, $K_{a,1}$, $K_{a,2}$, ΔH_1, ΔH_2) were allowed to vary freely.
